# First-in-Human Phase I Clinical Trial of SLC-391, a Novel and Selective AXL Inhibitor, in Patients with Advanced Solid Tumours

**DOI:** 10.3390/ph18121898

**Published:** 2025-12-17

**Authors:** Zaihui Zhang, Donna Morrison, Liang Lu, Madhu Singh, Jun Yan, Natasha Leighl, Scott A. Laurie, Sebastien Hotte

**Affiliations:** 1 SignalChem Lifesciences Corp., Richmond, BC V6V 2J1, Canada; donna_morrison@sinobiologicalus.com (D.M.); llu@signalchemcorp.com (L.L.); msingh@signalchemcorp.com (M.S.); jyan@signalchem.com (J.Y.); 2 Princess Margaret Cancer Centre, Toronto, ON M5G 2C4, Canada; natasha.leighl@uhn.ca; 3 The Ottawa Hospital Cancer Centre, Ottawa, ON K1H 8L6, Canada; slaurie@toh.ca; 4 Juravinski Cancer Centre, Hamilton, ON L8V 5C2, Canada; hotte@hhsc.ca

**Keywords:** AXL inhibitor, SLC-391, Phase I clinical trial, advanced solid tumours

## Abstract

**Background/Objectives:** AXL, a receptor tyrosine kinase of the TAM family, has emerged as a key target in cancer therapy due to its role in tumour growth, metastasis, immune evasion, and therapy resistance. SLC-391, a novel, orally bioavailable and selective AXL inhibitor, has demonstrated potent anti-tumour effects in preclinical studies. This first-in-human, open-label, multi-centre Phase I clinical trial (NCT03990454) was conducted to evaluate the safety, tolerability, pharmacokinetics (PK), and preliminary efficacy of SLC-391 in patients with advanced solid tumours. **Methods:** Using a 3 + 3 design, SLC-391 was administered orally, either once daily (from 25 mg up to 175 mg QD) or twice daily (from 75 mg to 200 mg BID) in 21-day cycles. **Results:** Following single and repeated dosing, SLC-391 was generally well tolerated by subjects. The maximum tolerated dose (MTD) was not reached in this study. A total of 34/35 subjects experienced at least one TEAE. Three (8.6%) subjects experienced Grade 3 TRAEs that were considered related to SLC-391. Eight SAEs were reported in five (14.3%) subjects (seven Grade 3 SAEs and one Grade 2 SAE), in 150 mg QD (3/6, 50%), 175 mg QD (1/2, 50%), and 110 mg BID (1/3, 33.3%) cohorts. Four SAEs in three (8.6%) subjects led to dose interruption, drug withdrawal, or study discontinuation. Three DLTs were reported in two subjects: one subject experienced Grade 3 hematochezia (SUSAR/DLT) at 175 mg QD, and another subject experienced Grade 3 thrombocytopenia associated with Grade 1 hematuria at 200 mg BID. The median T_max_ was 2.0 h. Plasma concentrations following multiple doses generally increased with higher doses and appeared to reach steady state by Day 21 and were generally dose-proportional. Twelve (12) out of 35 subjects with solid tumours achieved stable disease according to RECIST or mRECIST (mesothelioma), with durations of stable disease lasting up to 318 days on SLC-391 monotherapy. The clinical benefit rate was 34.3%. **Conclusions:** This first study of SLC-391 in adult subjects with advanced solid tumours demonstrated that a total daily dose of 300 mg (150 mg BID) of SLC-391 monotherapy was generally well tolerated, with no DLTs or SAEs observed at this dose. The drug’s promising safety profile, along with stable disease reported for several subjects with advanced solid tumours, provides a strong rationale for the phase 1b/2a clinical investigation of SLC-391 in combination with pembrolizumab in subjects with advanced or metastatic non-small cell lung cancer (NSCLC) (NCT05860296).

## 1. Introduction

AXL, a transmembrane receptor and a member of the TAM family of receptor tyrosine kinases, is implicated in many steps of the metastatic cascade and has emerged as a promising therapeutic target for many tumours, including lung cancer [1,2,3]. Upon activation, via ligand-dependent (Gas6) or ligand-independent mechanisms, AXL dimerizes, trans auto-phosphorylates, and activates several downstream signalling pathways, including PI3K-AKT-mTOR, MEK-ERK, p38, NF-κB, FAK, MAPK, and JAK/STAT, thus tightly controlling various cellular functions [1,3,4,5].

Several studies have suggested that AXL plays a vital role in promoting an immune-suppressive TME, which supports cancer progression, metastasis, and resistance to anticancer therapy [1,3,6,7,8,9]. AXL also appears to promote tumour immune evasion by downregulating DC, NK, and CD4+/CD8+ T-cells, decreasing major histocompatibility complex class I molecules, favouring immunosuppressive chemokines/cytokines, promoting myeloid-derived suppressor cell expansion, M1 to pro-tumour M2 macrophage polarisation, T-cell exclusion, activating regulatory T-reg cells, and increasing PD-L1 expression on tumour cells [2,7,8,9,10].

Further, elevated AXL expression has been shown to correlate with poor clinical prognosis in many solid and haematological tumours, including NSCLC, glioblastoma, sarcomas, and breast, colorectal, and GI cancers [3]. Growing evidence indicates that upregulated AXL expression promotes resistance to various therapies in NSCLC, acute myeloid leukaemia (AML), breast, colon, GI, head and neck, and other cancers [1,3,6].

SLC-391, 3-(5-[cyclopropylmethyl]-1,3,4-oxadiazol-2-yl)-5-(1-[piperidin-4-yl]-1*H*-pyrazol-4-yl)-pyridin-2-amine, is an orally bioavailable, potent, and selective small molecule inhibitor of AXL (IC_50_ of 9.6 nM against AXL, 42.3 nM against TYRO3, and 44 nM against MER). SLC-391 is an ATP analogue and functions as a type I kinase inhibitor by competing with ATP for binding at the ATP-binding pocket. By blocking ATP access, it prevents AXL autophosphorylation, which is required for its catalytic activation. It has been demonstrated that targeting AXL with SLC-391, alone or in combination with chemotherapy or immune checkpoint blockade, results in tumour growth inhibition in multiple solid tumour and haematological tumour models [11,12,13,14,15,16,17], including lung cancer, colon cancer, CML, and AML. Although a few selective small-molecule AXL inhibitors have been evaluated in clinical trials [11,18,19], no selective AXL inhibitor has been approved for the treatment of any type of cancer.

We report herein the findings of a multi-centre, open-label, first-in-human phase I dose escalation clinical study that investigated the safety and tolerability of SLC-391 in subjects with advanced solid tumours (NCT03990454) and established the recommended dose for future clinical investigations. Additionally, the study evaluated the PK and preliminary efficacy profiles of SLC-391.

## 2. Results

### 2.1. Patient Characteristics

A total of 35 subjects received at least one dose of SLC-391 in the study. Most subjects were white, male, and not Hispanic or Latino. The median age was 63 years, and the predominant primary tumour type was non-small cell lung cancer (45.7%), followed by esophageal (8.6%), hepatic (5.7%), and colorectal (5.7%) cancer. At study entry, most subjects had an ECOG performance status of 1. At screening, 85.7% of subjects had stage IV disease with adenocarcinoma (65.7%) as the dominant histological subtype. All subjects discontinued study treatment, and the most frequent reason for discontinuing study treatment was progressive disease (65.7%), followed by AEs (20.0%) and physician or subject decision to withdraw from the study (14.3%). The baseline demographics and clinical characteristics are summarised in Table 1.

In general, cancer disease characteristics were similar between QD (N = 20) and BID (N = 15) cohorts. The median duration since initial diagnosis was somewhat variable (QD: 30.8 months; BID: 39.0 months). Stage IV was the most common disease stage both at initial diagnosis (QD: 55.0%; BID: 60.0%) and at screening (QD: 85.0%; BID: 86.7%). Adenocarcinoma was the histological subtype in the majority of subjects across both QD (75.0%) and BID (53.3%) cohorts.

### 2.2. Drug Safety Evaluations

Drug exposure and DLTs: All 35 subjects enrolled in this study received at least one dose of study drug SLC-391 in their respective dose cohort. Twenty subjects received one of six escalating QD doses of SLC-391 (25 mg, 50 mg, 100 mg, 125 mg, 150 mg, and 175 mg), and 15 subjects received one of four escalating BID doses of SLC-391 (75 mg, 110 mg, 150 mg, and 200 mg).

Analysis of available PK data from QD cohorts suggested that a BID dosing schedule would provide less peak-to-trough variability, decrease the peak concentration at any given dose, and maintain more consistent SLC-391 concentrations; therefore, BID dosing was initiated with 75 mg. One subject in the 175 mg QD dose cohort (N = 2) experienced Grade 3 haematochezia, which was assessed as a DLT (suspected unexpected serious adverse reaction [SUSAR]). A second subject in the 200 mg BID dose cohort experienced Grade 3 thrombocytopenia associated with Grade 1 haematuria as a DLT. The 150 mg BID dose was well tolerated (N = 6) with no DLTs or SAEs.

For all subjects in the study (N = 35), the median total duration of study drug exposure (last dose date–first dose date + 1) was 63.0 days (range, 2–414 days). The compliance (median relative dose intensity) was 99.1%. Consistently, duration of exposure was similar between QD (N = 20) and BID (N = 15) cohorts (QD: 63.0 days vs. BID: 62.0 days). At the highest QD dose of 175 mg, the median duration of exposure was 10.5 days; only two subjects were enrolled in this dose cohort: one had an SAE and discontinued after receiving study drug for 2 days, and the other subject’s dose was interrupted during cycle 1, after which the subject discontinued the study. At the highest BID dose of 200 mg, the median duration of exposure was 28 days: one subject had a DLT and discontinued after receiving the study drug for 20 days; the second subject completed three cycles of dosing and discontinued due to progressive disease; and the third subject discontinued the study drug early in cycle 2 due to AEs. Median duration of exposure was the same (56.5 days) for the total daily dose of 150 mg (QD, N = 6) and 300 mg (150 mg BID, N = 6).

Adverse events: All 35 enrolled subjects received at least one dose of SLC-391, and 34/35 (97.1%) subjects experienced at least one treatment-emergent adverse event (TEAE). A total of 31 (88.6%) subjects had one or more treatment-related adverse event (TRAE) considered by the investigator to be related to SLC-391. Three (8.6%) subjects had a maximum of Grade 1, 19 (54.3%) subjects had a maximum of Grade 2, and 12 (34.3%) subjects had a maximum of Grade 3 TEAEs. Three (8.6%) subjects experienced Grade 3 TRAEs that were considered related to SLC-391. Two deaths were reported, both of which occurred after study drug discontinuation due to progressive disease. Eight SAEs were reported in five (14.3%) subjects (seven Grade 3 SAEs and one Grade 2 SAE), including Grade 3 possible TRAE haematochezia (SUSAR/DLT), and Grade 3 unrelated ascites, peritonitis, vomiting, lower GI haemorrhage, surgical intervention of sigmoid mass, and pelvic infection. An unrelated Grade 2 pleural effusion was reported for one subject. These events occurred in subjects across the following cohorts: 150 mg QD (3/6, 50%); 175 mg QD (1/2, 50%); and 110 mg BID (1/3, 33.3%). Four SAEs in three (8.6%) subjects led to dose interruption, drug withdrawal, or study discontinuation.

Dosing was interrupted in nine (25.7%) subjects due to TEAEs. Of these nine subjects, two (2/9, 22.2%) subjects continued in the study at a reduced dose. TEAEs leading to drug withdrawal or study discontinuation were reported in 20.0% (7/35) of subjects. Overall, a total of 19 (95.0%) subjects in QD cohorts and 15 (100.0%) subjects in BID cohorts experienced at least one TEAE. Subjects with TRAEs (90.0% vs. 86.7%), TEAEs leading to treatment or study discontinuation in subjects (20.0% vs. 20.0%), and subjects with Grade 3 TEAEs (30.0% vs. 40.0%) were comparable across the QD and BID cohorts. SAEs (20.0% vs. 6.7%) were higher in subjects administered QD doses compared with BID subjects. Overall, TEAEs leading to treatment interruption or missed dose (QD: 20.0% vs. BID: 40.0%) were higher in the BID subjects compared with the QD subjects. The TEAEs in BID cohorts were comparable to QD cohorts, even though the total daily doses were higher in BID cohort subjects. Treatment-emergent adverse events (TEAEs) occurring in ≥10.0% of subjects by SOC, PT, and maximum grade are summarised in Table 2.

At the highest dose of 200 mg BID (N = 3), most subjects reported nausea (100.0%), diarrhoea (66.7%), constipation (66.7%), fatigue (66.7%), decreased appetite (66.7%), headache (66.7%), and dysgeusia (66.7%), whereas at the second highest dose of 150 mg BID (N = 6), most subjects reported nausea (83.3%), fatigue (83.3%), diarrhoea (66.7%), constipation (66.7%), and dyspnoea (50.0%). More subjects (66.7% vs. 50.0%) reported Grade 3 TEAEs at the 200 mg BID dose compared with the 150 mg BID dose. TEAEs leading to drug withdrawal or study discontinuation were also higher in the 200 mg BID subjects compared with subjects at the 150 mg BID dose (66.7% vs. 16.7%).

Grade 3 GI TEAEs occurred in four subjects in the study: two subjects at 150 mg QD experienced ascites, intestinal mass, lower GI haemorrhage, nausea, and vomiting; one subject at 175 mg QD experienced haematochezia and abdominal pain upper; and one subject at 150 mg BID experienced diarrhoea. Study drug-related treatment-related adverse events (TRAEs) occurring in ≥10.0% of subjects by SOC and PT are summarised in Table 3.

### 2.3. Clinical Laboratory Evaluations

Most clinical chemistry-related toxicities were Grade 1 or 2, and they included hypokalaemia, hypomagnesaemia, hyponatraemia, hypophosphataemia, increased blood creatinine, and decreases in blood zinc, and they were all determined to be unrelated to SLC-391. There were no reports of heme toxicity or clinically significant abnormal ECG and neutrophil count changes, including QT interval or QTcF prolongation.

### 2.4. PK Analyses

PK parameters were calculated by standard non-compartmental methods for SLC-391. Due to the long half-life of SLC-391 compared to the dosing interval, it was not possible to calculate a number of PK parameters, including plasma half-life and rate of elimination. Accumulation was assessed from the mean accumulation ratio factor (AR factor) calculated as the repeated dose (C1D21) mean value/single dose (C1D1) mean value for the PK parameters C_max_, AUC_0–12_, and AUC_0–last_ for each dose group.

QD cohorts: Following single ascending oral doses (C1D1), SLC-391 was rapidly absorbed with median T_max_ values ranging from 0.9 to 2.1 h across the dose range of 25 mg to 175 mg QD. Mean C_max_ values increased with dose and ranged from 32.5 to 357.0 ng/mL. Similarly, mean AUC_0–last_ increased with dose from 269.5 to 1576.0 ng·h/mL.

**Figure 1:**
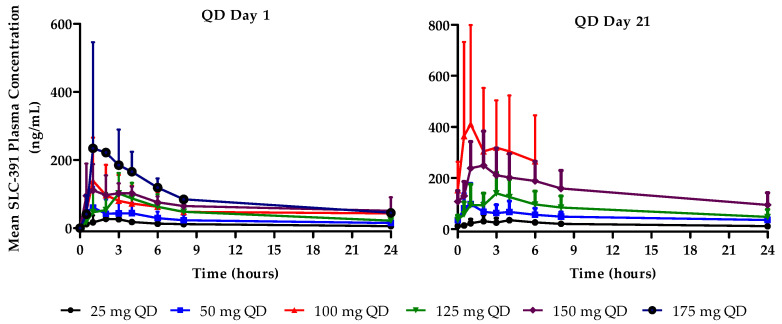
Mean plasma concentration–time profiles of SLC-391 on days 1 and 21 of cycle 1 following QD dosing.

Following multiple daily oral doses (C1D21), the median T_max_ values of SLC-391 ranged from 1.5 to 2.1 h across the dose range of 25 mg to 150 mg QD. C1D21 PK results are not available for the 175 mg QD dose cohort as the subjects discontinued the study prior to C1D21. Mean C_max_ values generally increased with dose and ranged from 41.9 to 436.5 ng/mL. Similarly, mean AUC_0–last_ generally increased with dose from 461.3 to 3620.0 ng·h/mL. C_min_ values ranged from 10.3 to 151.8 ng/mL after 21 days of QD dosing. The AR factors for C_max_, AUC_0–last_, and AUC_0–12_ generally increased after 21-day dosing of SLC-391 across all doses. Mean plasma concentration–time profiles are shown in Figure 1, and the C1D1 and C1D21 PK parameters are summarised in Appendix B for the QD cohorts.

BID cohorts: Following single ascending oral doses (C1D1), SLC-391 was rapidly absorbed with median T_max_ values ranging from 2.0 to 3.1 h across the dose range of 75 mg to 200 mg BID. Mean C_max_ values generally increased with dose and ranged from 95.2 to 235.8 ng/mL. Similarly, mean AUC_0–last_ increased from 1172.3 to 2328.3 ng·h/mL across the dose range.

**Figure 2:**
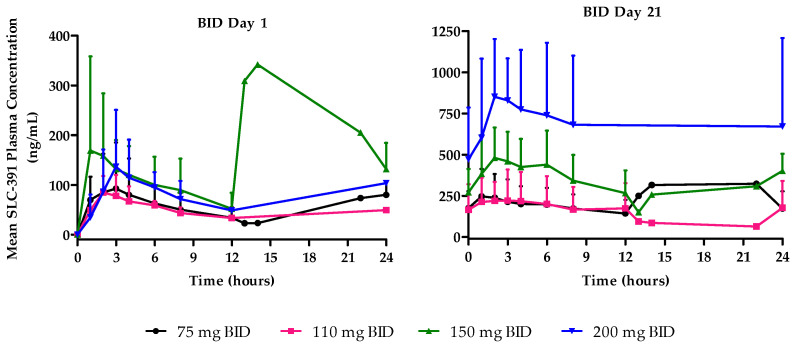
Mean plasma concentration–time profiles of SLC-391 on days 1 and 21 of cycle 1 following BID dosing.

Following multiple oral BID doses (C1D21), the median T_max_ values of SLC-391 ranged from 1.9 to 2.9 h across the dose range of 75 mg to 200 mg BID. Mean C_max_ values generally increased with dose and ranged from 248.7 to 874.5 ng/mL. Similarly, mean AUC_0–last_ increased from 4280.0 to 16,405.0 ng·h/mL. C_min_ values ranged from 145.6 to 471.5 ng/mL after 21 days of BID dosing and generally increased with dose. The AR factors for C_max_, AUC_0–last_, and AUC_0–12_ generally increased after 21-day dosing of SLC-391 across all doses. Mean plasma concentration–time profiles are shown in Figure 2, and the C1D1 and C1D21 PK parameters are summarised in Appendix C for the BID cohorts.

### 2.5. Efficacy Analysis

For the 35 subjects treated at any dose of SLC-391, all subjects had measurable disease at baseline and were evaluable for efficacy by RECIST 1.1. Subjects with mesothelioma (4/4), NSCLC (4/16), mammary adenocarcinoma (1/1), appendix adenocarcinoma (1/1), melanoma (1/1), and head and neck cancer (1/1) had a best overall response (BOR) of stable disease with durations ranging from 44 days to 318 days. Amongst the BID cohorts, a better rate for stable disease was noted in the 150 mg BID (50.0%, 3/6) subjects. The best percent change in target lesion diameter was observed in one subject each at 50 mg QD (26.7% decrease at cycle 7), 150 mg QD (10.7% decrease at cycle 7), 110 mg BID (22.2% decrease on day 275), and 150 mg BID (8.9% decrease at the end of treatment visit). Results showed that 12 of 35 subjects had stable disease as per RECIST 1.1 or modified RECIST (mesothelioma).

## 3. Discussion

This first-in-human study determined the safety and MTD of SLC-391 administered orally, either QD or BID in 21-day cycles, to subjects with advanced solid tumours. Additionally, the study evaluated the PK and preliminary efficacy profile of SLC-391.

The data from this study demonstrate that a total daily dose of 300 mg (150 mg BID) of SLC-391 monotherapy was generally well tolerated, with no DLTs or SAEs observed at this dose. The incidence of the most common TEAEs, regardless of severity or relationship to SLC-391, was generally consistent across subjects in QD and BID cohorts and included nausea, fatigue, diarrhoea, and vomiting. Similar GI toxicity has been observed with other AXL inhibitors/agents. Proactive management with anti-emetic and anti-diarrheal medications given before or at the start of SLC-391 dosing can be part of the strategy to mitigate these GI adverse effects. Three DLTs were reported in two subjects: one subject experienced Grade 3 hematochezia (SUSAR/DLT) at 175 mg QD, and another subject experienced Grade 3 thrombocytopenia associated with Grade 1 hematuria at 200 mg BID. Eight SAEs were reported in five subjects, including Grade 3 possibly related haematochezia, and Grade 3 unrelated ascites, peritonitis, vomiting, lower GI haemorrhage, surgical intervention of sigmoid mass, and pelvic infection. An unrelated Grade 2 pleural effusion was reported for one subject. The maximum tolerated dose (MTD) was not reached in this study. However, based on the safety profile, including dose-limiting thrombocytopenia observed in one of three subjects in the 200 mg BID group and the SLC-391 PK data, and in vitro/in vivo efficacy data, the safety review committee recommended a 150 mg BID dose for further investigations. No DLT or SAEs were reported for subjects who received the 150 mg BID dose.

Pharmacokinetic results, after administration of single and repeated doses of SLC-391 for both QD and BID dosing regimens, indicated that SLC-391 was quickly absorbed into the systemic circulation. Plasma concentrations following multiple doses generally increased with higher doses and appeared to reach a steady state by day 21. The exploratory analysis on dose proportionality indicated dose-dependent trends in C_max_, C_min_, AUC_0–12_, and AUC_0–last_, suggesting approximately dose-proportional increases in exposure within the range of the doses tested after single and repeated doses. In addition, for the total daily dose of 150 mg (75 mg BID vs. 150 mg QD), the BID dosing schedule provided less peak-to-trough variability and maintained more consistent SLC-391 concentrations. A cross-board increase in AR factors for C_max_, AUC_0–12_, and AUC_0–last_ indicated a moderate accumulation of SLC-391 after repeated dosing. For the QD dosing regimen, all AR factors were in the range of 1.3 to 3.1, indicating a moderate accumulation. However, the BID dosing regimen demonstrated a more pronounced accumulation of SLC-391 after repeated dosing with all AR factors ranging from 2.2 to 8.7, with the highest accumulation in the 200 mg BID cohort. This accumulation of SLC-391 may have contributed to the more pronounced adverse events experienced by the patients in the 200 mg BID cohort.

Although the efficacy profile of SLC-391 was not definitively established due to the phase of development, several subjects had stable disease and clinical benefit. Notably, within the BID cohorts, the 150 mg BID subjects (3/6, 50.0%) had a higher rate of stable disease, which is promising and suggests that achieving and maintaining sufficiently high and sustained exposure to SLC-391 may enhance efficacy. In the study, 12 of 35 subjects with solid tumours achieved stable disease according to RECIST or mRECIST (mesothelioma), with durations of stable disease lasting up to 318 days on SLC-391 monotherapy. The clinical benefit rate was 34.3%.

It is worth noting that six patients showed a prolonged period of stable disease for more than half a year following SLC-391 monotherapy, even though they all had a stage IV diagnosis and a history of different prior cancer treatments. The longest duration of stable disease was 318 days. The presence of stable disease for a prolonged period of time in some patients after SLC-391 monotherapy, especially as the patient population was heavily pretreated and refractory to multiple therapies, was encouraging and warrants further clinical development of SLC-391.

## 4. Materials and Methods

### 4.1. Study Objectives and Ethics

The primary objectives of this phase I study were to evaluate the safety of SLC-391, administered orally once daily (QD) or twice daily (BID), in patients with previously treated, advanced solid tumours and to determine the maximum tolerated dose (MTD) of SLC-391. The secondary objectives were to characterise the pharmacokinetics (PK) profile of SLC-391, to determine the recommended phase 2 dose (RP2D), and to preliminarily evaluate the efficacy of SLC-391. The protocol, protocol amendments, informed consent form (ICF), Investigator’s Brochure, and other relevant documents were submitted to a research ethics board (REB) by the investigators, and they were reviewed and approved by the REB at Princess Margaret Cancer Centre (Toronto, ON, Canada), the Ottawa Hospital Cancer Centre (Ottawa, ON, Canada) and the Juravinski Cancer Centre (Hamilton, ON, Canada) before the study was initiated and before any amendments were implemented. This study was performed in full compliance with the International Conference on Harmonization (ICH) Technical Requirements for Registration of Pharmaceuticals for Human Use and with all applicable local good clinical practice (GCP) regulations. All patients provided written informed consent.

### 4.2. Eligibility

This study enrolled male and female subjects aged 18 years or older who had a histologically or cytologically confirmed diagnosis of a solid tumour malignancy that was advanced and/or metastatic or unresectable, and for which standard curative measures did not exist or were no longer effective. Subjects were required to have measurable disease as per Response Evaluation Criteria in Solid Tumours (RECIST) 1.1 [20,21] or as per a modified RECIST (mRECIST), when applicable. Certain health and life expectancy, and sufficient elapsed time from prior cancer therapy, were also required. Subjects were excluded from the study if they had previously received an AXL inhibitor, had prostate cancer and were concurrently receiving abiraterone or enzalutamide, had active central nervous system metastases and/or carcinomatous meningitis, had certain other health conditions, or had received a prior solid organ or bone marrow transplant. A full list of inclusion and exclusion criteria is found in Appendix A.

### 4.3. Drug Supply

SLC-391 drug product was formulated as a solid oral formulation of hard gelatin capsules filled with the SLC-391 drug substance and excipients (lactose monohydrate, microcrystalline cellulose, and magnesium stearate), and was manufactured in three strengths: 10 mg, 25 mg, and 50 mg. SLC-391 capsules were packaged in round, white, and opaque high-density polyethylene bottles with child-resistant polypropylene caps and induction-sealed aluminium liners, and they were labelled with investigational use statements in accordance with local regulations (25 capsules per bottle). Bottles of SLC-391 were stored at a controlled room temperature of 15–25 °C and accessed/dispensed only by personnel authorised for the applicable clinical study.

### 4.4. Study Design, Dose Selection, and Dose Escalation

**Figure 3:**
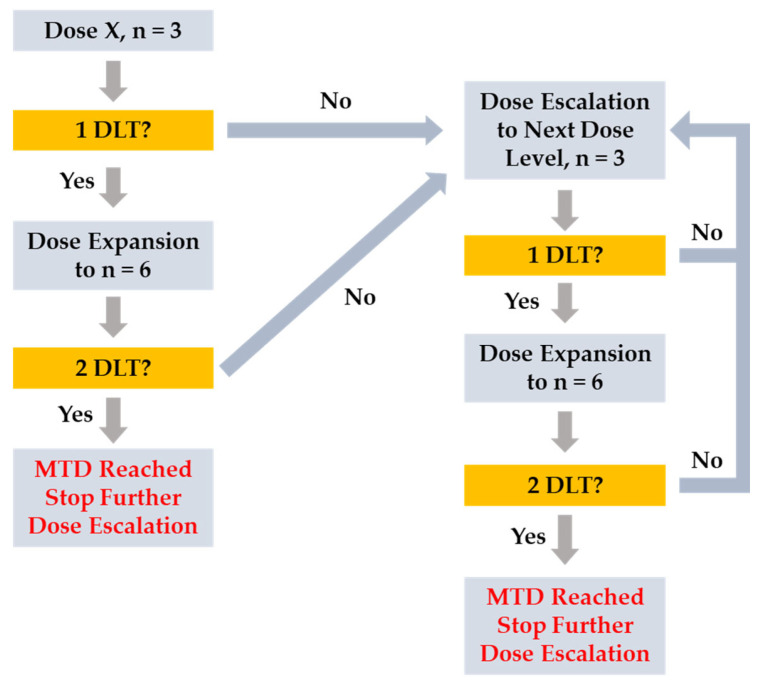
Dose escalation flowchart.

This study employed a standard 3 + 3 dose escalation design [22] with a starting dose of 25 mg QD. Evaluation of a dose cohort of at least 3 subjects who had completed cycle 1 (21-day cycle) was required prior to defining a new SLC-391 dose and opening the next dose cohort. Dose escalation decisions took into account all available data from evaluable subjects as well as PK and the safety profile of prior dose cohorts. A treatment cycle was defined as 21 continuous calendar days of SLC-391 dosing, regardless of whether the study drug was interrupted. There was a minimum 5-day stagger between dosing of the first and second subjects in each dose cohort. Dose escalation decisions were made by a data review committee (DRC) composed of, at minimum, the medical monitor, at least one principal investigator, and a sponsor representative, per the study DRC Plan. Dose escalation could continue until 2 or more subjects in the same dose cohort experienced a dose-limiting toxicity (DLT). DLTs were defined as Grade 3 or 4 adverse drug reactions (ADRs), with grades as defined in the National Cancer Institute Common Toxicity Criteria for Adverse Events (NCI-CTCAE) version 5.0. To be eligible for DLT evaluation, the subject would have to take at least 75% of the doses in cycle 1 or have stopped dosing due to toxicity for each cohort. The MTD was defined as the highest dose at which no more than 1 of 6 subjects experienced a DLT during cycle 1 of the study drug. The MTD would have been exceeded if 2 or more subjects in a dose cohort of up to 6 subjects experienced a DLT. Subjects could continue treatment until disease progression, unacceptable toxicity, death, withdrawal of consent, or loss to follow-up. Subjects were followed for 30 days after the last dose of SLC-391 or until initiating any other anticancer therapy. The dose escalation flowchart is shown in Figure 3.

### 4.5. PK Analyses

During QD dosing, blood samples for determination of SLC-391 plasma concentrations were collected predose and at 0.5, 1, 2, 3, 4, 6, 8, and 24 h after SLC-391 administration on cycle 1 day 1 (C1D1) and cycle 1 day 21 (C1D21). During BID dosing, blood samples were collected predose and at 1, 2, 3, 4, 6, 8, and 12 h after the first daily dose (before the second daily dose). Where possible, further samples were then collected at 13, 14, 22, and 24 h after the initial dose (predose C1D2). Blood samples were also collected on C1D21 (steady state) predose and at 1, 2, 3, 4, 6, 8, and 12 h after the first daily dose (before the second daily dose). Where possible, further samples were then collected at 13, 14, 22, and 24 h after the initial C1D21 dose (predose C2D1). An additional blood sample was also taken, where possible, at the end of treatment (EOT) visit.

After collection, blood samples were centrifuged, and plasma was separated and stored at −80 °C. Plasma concentrations of SLC-391 were measured by a validated analytical method involving liquid–liquid extraction followed by high-performance liquid chromatography (HPLC)–mass/mass spectroscopy assay using a Water Quattro Premier tandem quadrupole mass spectroscopy with Waters MassLynx data acquisition and processing software. Plasma analysis was carried out by Microconstants (San Diego, CA, USA) in compliance with the US FDA GLP regulations. The calibration range of the assay was from 0.100 to 100 ng/mL. Quality control samples prepared at 4 different analyte concentrations were analysed with each batch of samples against a separately prepared calibration standard. PK parameters were collected following single (day 1) and repeat dose (day 21) administration. PK parameters were summarised with appropriate descriptive statistics where possible (e.g., mean, geometric mean, median, maximum, minimum, and coefficient of variation). Descriptive PK parameters were determined by standard non-compartmental methods based on the concentration–time data of each subject. The nominal time was used for descriptive statistics calculations, and the actual sample collection times were used in PK analyses. PK analyses were performed using validated Phoenix WinNonlin Professional 8.3 (Certara, L.P., St. Louis, MO, USA).

### 4.6. Patient Evaluation on Treatment

Before enrolment in the study, all patients received a detailed medical history and physical examination. Clinical laboratory evaluations included clinical chemistry, complete blood count, urinalysis, coagulation factors, and a lipid panel. Other safety assessments included vital signs, physical examination, electrocardiogram, and echocardiogram with multigated acquisition (ECHO/MUGA). Safety assessments were performed on days 1, 8, 15, and 21 of cycle 1, and on day 1 of cycles 2 to 6.

The safety of SLC-391 was evaluated by means of dose-limiting toxicities (DLTs), adverse events (AEs), Eastern Cooperative Oncology Group (ECOG) performance status, physical examinations, vital signs, electrocardiogram (ECG), and laboratory test results.

Disease assessments were performed according to RECIST 1.1 [20,21] and included imaging and physical examination as appropriate for disease type and location. Disease assessments were completed at screening (within 30 days of C1D1) and every 3 cycles (±7 days) relative to C1D1 until objective disease progression, study drug discontinuation, or withdrawal of informed consent.

## 5. Conclusions

In conclusion, this first study of SLC-391 in adult subjects with solid tumours demonstrated that a total daily dose of 300 mg (150 mg BID) of SLC-391 monotherapy was generally well tolerated, with no DLTs or SAEs observed at this dose. The drug’s promising safety profile, along with stable disease reported for several subjects with advanced cancer in addition to clinical benefit, warrants future clinical investigations. The overall risk to study participants appears to be acceptable, particularly given the lack of effective alternative treatments for patients with advanced cancer.

## Figures and Tables

**Table 1:** Baseline demographics and clinical characteristics.

Characteristic	Overall QD (N = 20)	Overall BID (N = 15)	Overall Study (N = 35)
Age, median (range) (y)	63.5 (38–78)	63.0 (33–81)	63.0 (33–81)
Sex			
Male	13 (65.0)	10 (66.7)	23 (65.7)
Female	7 (3.0)	5 (33.3)	12 (34.3)
Race			
Asian	2 (10.0)	1 (6.7)	3 (8.6)
White	18 (90.0)	12 (80.0)	30 (85.7)
Black or African American	0	1 (6.7)	1 (2.9)
Native Hawaiian or other Pacific Islander	0	1 (6.7)	1 (2.9)
Ethnicity, n %			
Not Hispanic or Latino	20 (100.0)	15 (100.0)	35 (100.0)
Hispanic or Latino	0	0	0
Baseline ECOG, n (%)			
0	6 (30.0)	6 (40.0)	12 (34.3)
1	14 (70.0)	9 (60.0)	23 (65.7)
Primary tumour type			
Colon/rectum	2 (10.0)	0	2 (5.7)
Endometrium/uterus	1 (5.0)	0	1 (2.9)
Oesophagus	0	3 (20.0)	3 (8.6)
Head and neck	0	1 (6.7)	1 (2.9)
Liver	0	2 (13.3)	2 (5.7)
Melanoma	0	1 (6.7)	1 (2.9)
Non-small cell lung	10 (50.0)	6 (40.0)	16 (45.7)
Ovary	0	1 (6.7)	1 (2.9)
Pancreas	1 (5.0)	0	1 (2.9)
Prostate	1 (5.0)	0	1 (2.9)
Mesothelioma	2 (5.0)	0	2 (5.8)
Appendix	1 (5.0)	0	1 (2.9)
Mammary analogue secretory adenocar-cinoma	1 (5.0)	0	1 (2.9)
Peritoneal mesothelioma	1 (5.0)	0	1 (2.9)
Pleural epithelioid mesothelioma	0	1 (6.7)	1 (2.9)
Prior chemotherapies			
Median number of treatment	3.0	4.0	3.0

**Table 2:** Summary of treatment-emergent adverse events (TEAEs) occurring in ≥10.0% of subjects by SOC, PT and maximum grade.

System Organ Class (SOC) Preferred Term (PT)	CTCAE Grade ^a^	Overall QD (N = 20) n (%)	Overall BID (N = 15) n (%)	Overall Study (N = 35) n (%)
Any treatment-emergent adverse event (TEAE)	All	19 (95.0)	15 (100.0)	34 (97.1)
	1	1 (5.0)	2 (13.3)	3 (8.6)
	2	12 (60.0)	7 (46.7)	19 (54.3)
	3	6 (30.0)	6 (40.0)	12 (34.3)
Gastrointestinal disorders	All	17 (85.0)	13 (86.7)	30 (85.7)
	1	8 (40.0)	7 (46.7)	15 (42.9)
	2	6 (30.0)	5 (33.3)	11 (31.4)
	3	3 (15.0)	1 (6.7)	4 (11.4)
Nausea	All	13 (65.0)	11 (73.3)	24 (68.6)
	1	8 (40.0)	7 (46.7)	15 (42.9)
	2	4 (20.0)	4 (26.7)	8 (22.9)
	3	1 (5.0)	0	1 (2.9)
Diarrhoea	All	11 (55.0)	9 (60.0)	20 (57.1)
	1	8 (40.0)	6 (40.0)	14 (40.0)
	2	3 (15.0)	2 (13.3)	5 (14.3)
	3	0	1 (6.7)	1 (2.9)
Vomiting	All	11 (55.0)	6 (40.0)	17 (48.6)
	1	8 (40.0)	5 (33.3)	13 (37.1)
	2	2 (10.0)	1 (6.7)	3 (8.6)
	3	1 (5.0)	0	1 (2.9)
Constipation	All	7 (35.0)	7 (46.7)	14 (40.0)
	1	5 (25.0)	5 (33.3)	10 (28.6)
	2	2 (10.0)	2 (13.3)	4 (11.4)
Abdominal distension	All	3 (15.0)	3 (20.0)	6 (17.1)
	1	2 (10.0)	2 (13.3)	4 (11.4)
	2	1 (5.0)	1 (6.7)	2 (5.7)
Abdominal pain	All	5 (25.0)	0	5 (14.3)
	1	4 (20.0)	0	4 (11.4)
	2	1 (5.0)	0	1 (2.9)
General disorders and administration site conditions	All	15 (75.0)	12 (80.0)	27 (77.1)
	1	7 (35.0)	5 (33.3)	12 (34.3)
	2	8 (40.0)	6 (40.0)	14 (40.0)
	3	0	1 (6.7)	1 (2.9)
Fatigue	All	12 (60.0)	10 (66.7)	22 (62.9)
	1	6 (30.0)	4 (26.7)	10 (28.6)
	2	6 (30.0)	5 (33.3)	11 (31.4)
	3	0	1 (6.7)	1 (2.9)
Chills	All	2 (10.0)	4 (26.7)	6 (17.1)
	1	2 (10.0)	4 (26.7)	6 (17.1)
Pyrexia	All	3 (15.0)	2 (13.3)	5 (14.3)
	1	2 (10.0)	2 (13.3)	4 (11.4)
	2	1 (5.0)	0	1 (2.9)
Oedema peripheral	All	2 (10.0)	2 (13.3)	4 (11.4)
	1	2 (10.0)	1 (6.7)	3 (8.6)
	2	0	1 (6.7)	1 (2.9)
Respiratory, thoracic, and mediastinal disorders	All	11 (55.0)	8 (53.3)	19 (54.3)
	1	3 (15.0)	2 (13.3)	5 (14.3)
	2	6 (30.0)	6 (40.0)	12 (34.3)
	3	2 (10.0)	0	2 (5.7)
Dyspnoea	All	7 (35.0)	5 (33.3)	12 (34.3)
	1	2 (10.0)	0	2 (5.7)
	2	4 (20.0)	5 (33.3)	9 (25.7)
	3	1 (5.0)	0	1 (2.9)
Cough	All	4 (20.0)	3 (20.0)	7 (20.0)
	1	4 (20.0)	2 (13.3)	6 (17.1)
	2	0	1 (6.7)	1 (2.9)
Nervous system disorders	All	10 (50.0)	5 (33.3)	15 (42.9)
	1	8 (40.0)	3 (20.0)	11 (31.4)
	2	2 (10.0)	2 (13.3)	4 (11.4)
Headache	All	2 (10.0)	3 (20.0)	5 (14.3)
	1	2 (10.0)	3 (20.0)	5 (14.3)
Dizziness	All	4 (20.0)	0	4 (11.4)
	1	4 (20.0)	0	4 (11.4)
Dysgeusia	All	2 (10.0)	2 (13.3)	4 (11.4)
	1	1 (5.0)	0	1 (2.9)
	2	1 (5.0)	2 (13.3)	3 (8.6)
Metabolism and nutrition disorders	All	9 (45.0)	5 (33.3)	14 (40.0)
	1	5 (25.0)	0	5 (14.3)
	2	2 (10.0)	5 (33.3)	7 (20.0)
	3	2 (10.0)	0	2 (5.7)
Decreased appetite	All	7 (35.0)	5 (33.3)	12 (34.3)
	1	6 (30.0)	1 (6.7)	7 (20.0)
	2	1 (5.0)	4 (26.7)	5 (14.3)
Musculoskeletal and connective tissue disorders	All	8 (40.0)	6 (40.0)	14 (40.0)
	1	5 (25.0)	2 (13.3)	7 (20.0)
	2	2 (10.0)	3 (20.0)	5 (14.3)
	3	1 (5.0)	1 (6.7)	2 (5.7)
Back pain	All	2 (10.0)	2 (13.3)	4 (11.4)
	1	1 (5.0)	0	1 (2.9)
	2	1 (5.0)	2 (13.3)	3 (8.6)
Infections and infestations	All	4 (20.0)	7 (46.7)	11 (31.4)
	1	0	1 (6.7)	1 (2.9)
	2	3 (15.0)	5 (33.3)	8 (22.9)
	3	1 (5.0)	1 (6.7)	2 (5.7)
Urinary tract infection	All	1 (5.0)	3 (20.0)	4 (11.4)
	2	1 (5.0)	3 (20.0)	4 (11.4)
Skin and subcutaneous tissue disorders	All	7 (35.0)	3 (20.0)	10 (28.6)
	1	4 (20.0)	2 (13.3)	6 (17.1)
	2	3 (15.0)	0	3 (8.6)
	3	0	1 (6.7)	1 (2.9)
Investigations	All	5 (25.0)	3 (20.0)	8 (22.9)
	1	5 (25.0)	2 (13.3)	7 (20.0)
	2	0	1 (6.7)	1 (2.9)
Weight decreased	All	4 (20.0)	2 (13.3)	6 (17.1)
	1	4 (20.0)	2 (13.3)	6 (17.1)
Renal and urinary disorders	All	1 (5.0)	5 (33.3)	6 (17.1)
	1	0	3 (20.0)	3 (8.6)
	2	1 (5.0)	1 (6.7)	2 (5.7)
	3	0	1 (6.7)	1 (2.9)
Haematuria	All	1 (5.0)	4 (26.7)	5 (14.3)
	1	1 (5.0)	3 (20.0)	4 (11.4)
	2	0	1 (6.7)	1 (2.9)
Psychiatric disorders	All	2 (10.0)	3 (20.0)	5 (14.3)
	1	2 (10.0)	1 (6.7)	3 (8.6)
	2	0	2 (13.3)	2 (5.7)
Vascular disorders	All	3 (15.0)	2 (13.3)	5 (14.3)
	2	2 (10.0)	1 (6.7)	3 (8.6)
	3	1 (5.0)	1 (6.7)	2 (5.7)
Blood and lymphatic system disorders	All	2 (10.0)	2 (13.3)	4 (11.4)
	1	0	1 (6.7)	1 (2.9)
	2	1 (5.0)	0	1 (2.9)
	3	1 (5.0)	1 (6.7)	2 (5.7)

^a^ CTCAE Grade: Grade 1 = Mild; Grade 2 = Moderate; Grade 3 = Severe.

**Table 3:** Summary of treatment-related adverse events (TRAEs) occurring in ≥10.0% of subjects by SOC and PT.

System Organ Class (SOC) Preferred Term (PT)	Relatedness	Overall QD (N = 20) n (%)	Overall BID (N = 15) n (%)	Overall Study (N = 35) n (%)
Any treatment-related adverse event (TRAE ^a^)	All	18 (90.0%)	13 (86.7%)	31 (88.6%)
	Possibly	8 (40.0%)	7 (46.7%)	15 (42.9%)
	Probably	3 (15.0%)	1 (6.7%)	4 (11.4%)
	Definitely	7 (35.0%)	5 (33.3%)	12 (34.3%)
Gastrointestinal disorders	All	16 (80.0%)	11 (73.3%)	27 (77.1%)
	Possibly	8 (40.0%)	5 (33.3%)	13 (37.1%)
	Probably	2 (10.0%)	1 (6.7%)	3 (8.6%)
	Definitely	6 (30.0%)	5 (33.3%)	11 (31.4%)
Nausea	All	11 (55.0%)	8 (53.3%)	19 (54.3%)
	Possibly	5 (25.0%)	4 (26.7%)	9 (25.7%)
	Probably	2 (10.0%)	0	2 (5.7%)
	Definitely	4 (20.0%)	4 (26.7%)	8 (22.9%)
Diarrhoea	All	10 (50.0%)	7 (46.7%)	17 (48.6%)
	Possibly	5 (25.0%)	4 (26.7%)	9 (25.7%)
	Probably	0	1 (6.7%)	1 (2.9%)
	Definitely	5 (25.0%)	2 (13.3%)	7 (20.0%)
Vomiting	All	9 (45.0%)	4 (26.7%)	13 (37.1%)
	Possibly	3 (15.0%)	1 (6.7%)	4 (11.4%)
	Probably	1 (5.0%)	0	1 (2.9%)
	Definitely	5 (25.0%)	3 (20.0%)	8 (22.9%)
General disorders and administration site conditions	All	6 (30.0%)	4 (26.7%)	10 (28.6%)
	Possibly	3 (15.0%)	3 (20.0%)	6 (17.1%)
	Definitely	3 (15.0%)	1 (6.7%)	4 (11.4%)
Fatigue	All	5 (25.0%)	4 (26.7%)	9 (25.7%)
	Possibly	2 (10.0%)	3 (20.0%)	5 (14.3%)
	Definitely	3 (15.0%)	1 (6.7%)	4 (11.4%)
Metabolism and nutrition disorders	All	6 (30.0%)	3 (20.0%)	9 (25.7%)
	Possibly	4 (20.0%)	1 (6.7%)	5 (14.3%)
	Definitely	2 (10.0%)	2 (13.3%)	4 (11.4%)
Decreased appetite	All	5 (25.0%)	3 (20.0%)	8 (22.9%)
	Possibly	3 (15.0%)	1 (6.7%)	4 (11.4%)
	Definitely	2 (10.0%)	2 (13.3%)	4 (11.4%)
Skin and subcutaneous tissue disorders	All	6 (30.0%)	2 (13.3%)	8 (22.9%)
	Possibly	4 (20.0%)	2 (13.3%)	6 (17.1%)
	Probably	2 (10.0%)	0	2 (5.7%)
Nervous system disorders	All	4 (20.0%)	2 (13.3%)	6 (17.1%)
	Possibly	1 (5.0%)	1 (6.7%)	2 (5.7%)
	Probably	2 (10.0%)	0	2 (5.7%)
	Definitely	1 (5.0%)	1 (6.7%)	2 (5.7%)
Dysgeusia	All	2 (10.0%)	2 (13.3%)	4 (11.4%)
	Possibly	0	1 (6.7%)	1 (2.9%)
	Probably	1 (5.0%)	0	1 (2.9%)
	Definitely	1 (5.0%)	1 (6.7%)	2 (5.7%)

^a^ TRAEs include TRAEs that were possibly related, probably related, and definitely related to SLC-391.

## Data Availability

The original contributions presented in this study are included in the article. Further inquiries can be directed to the corresponding author.

## Abbreviations

The following abbreviations are used in this manuscript:

AD	Adverse event.
AR	Accumulation ratio.
AUC_0–12_	Area under the concentration–time curve from time zero to 12 h.
AUC_0–last_	Area under the curve extrapolated to the last measured time point.
BID	Twice daily.
C_max_	Maximum plasma concentration.
C_min_	Minimum plasma concentration.
CR	Complete response.
DLT	Dose-limiting toxicity.
DRC	Data review committee.
ECG	Electrocardiogram.
ECOG	Eastern Cooperative Oncology Group.
ICF	Informed consent form.
ICH	International Conference on Harmonization.
MTD	Maximum tolerated dose.
NCI-CTCA	National Cancer Institute Common Terminology Criteria for Adverse Events.
NSCLC	Non-small cell lung cancer.
PD	Progressive disease.
PK	Pharmacokinetics.
PR	Partial response.
QD	Once daily.
QTcF	Fridericia heart rate–corrected QT interval.
REB	Research Ethics Board.
RECIST	Response Evaluation Criteria in Solid Tumours.
RP2D	Recommended phase 2 dose.
SAE	Serious adverse event.
SD	Stable disease.
SUSAR	Suspected Unexpected Serious Adverse Reaction.
TEAE	Treatment-emergent adverse event.
TRAE	Treatment-related adverse event.
T_max_	Time of maximum plasma concentration.
TME	Tumour microenvironment.

## Appendix A. Inclusion and Exclusion Criteria

- Inclusion Criteria

This study enrolled male and female subjects aged 18 years or older who had a histologically or cytologically confirmed diagnosis of a solid tumour malignancy that was advanced and/or metastatic or unresectable and for which standard curative measures did not exist or were no longer effective.

Subjects eligible to participate in this study had to meet all of the following criteria:

Be 18 years of age or older at the time of signing the informed consent.

Have a histologically or cytologically confirmed diagnosis of a solid tumour malignancy that is advanced and/or metastatic or unresectable, and for which standard or curative measures do not exist or are no longer effective.

Have measurable disease as per RECIST 1.1 or as per a modified RECIST, if applicable.

Have a performance status score of 0 or 1 according to the ECOG scale.

Be able to ingest oral medication.

Have adequate organ function, as defined in the following table.

**System**	**Parameter**	**Laboratory Values**
Hematologic	Absolute neutrophil count	≥1.5 × 10^9^/L (1500 cells/mm^3^) (without need for growth factor supplementation)
	White blood cell count	≥Lower limit of normal range and ≤2.0 × upper limit of normal range (ULN) (an aetiology for any values > ULN must be documented to rule out infection)
	Haemoglobin	≥90 g/L
	Platelets	≥75 × 10^9^/L
	Partial thromboplastin time	≤1.5 × ULN
	Prothrombin time and/or international normalised ratio	≤1.5 × ULN
Hepatic	Total bilirubin	≤1.5 × ULN
	Aspartate aminotransferase and alanine aminotransferase	≤2.5 × ULN, or ≤5 × ULN if liver lesions present (i.e., liver metastasis or primary tumour of the liver for hepatocellular carcinoma)
Renal	Creatinine OR Creatinine clearance	<1.5 × ULN ≥60 mL/min

Have recovered to ≤Grade 1 from the effects of any prior cancer therapy, except for alopecia; irreversible neuropathy should have recovered to ≤Grade 2. Toxic effects also include laboratory test abnormalities.

Be afebrile at baseline prior to SLC-391 administration (i.e., <38 °C).

Have a life expectancy greater than 3 months, in the investigator’s opinion.

The following time must have elapsed between the previous therapy for cancer and the first dose of SLC-391:

At least 4 weeks since previous cancer-directed therapy, including investigational agents or devices (cytotoxic agents, targeted therapy including monoclonal antibodies, immunotherapy, hormonal therapy, and prior radiotherapy), with the exception of nitrosoureas/mitomycin, where 6 weeks since these therapies must have elapsed.

At least 4 weeks or five times the elimination half-life (whichever is shorter) of any investigational agents, including drugs, biologics, or combination products.

At least 4 weeks since any major surgery.

Sexually active women of childbearing potential and sexually active male subjects with a female partner of childbearing potential or pregnant must agree to use acceptable methods of contraception to avoid pregnancy from screening, for the duration of the study, and for 3 months after the last dose of study drug. Male subjects must also agree to refrain from donating sperm for the duration of the study, including during dose interruptions and for 3 months after the last dose administered.

Be able and willing to provide signed informed consent and comply with the requirements, assessment schedule, dosing schedule, and restrictions listed in the ICF and study protocol.

B. Exclusion Criteria

Subjects who met any of the following criteria were ineligible for the study:

Prior use of any AXL inhibitor.

Localised or metastatic prostate cancer subjects who are concurrently receiving abiraterone or enzalutamide. Those subjects on stable (>3 months) anticancer hormonal therapy are allowed.

Refractory nausea and vomiting, chronic GI diseases, GI bleeding, ulceration, or perforation within 12 weeks prior to the first dose of the study drug, or significant bowel resection that would preclude adequate absorption, distribution, metabolism, or excretion of the study drug.

History of myocardial infarction, unstable angina, congestive heart failure (New York Heart Association class ≥ III/IV), cerebrovascular accident, transient ischaemic attack, limb claudication at rest, stroke, or subarachnoid haemorrhage, coagulopathy, deep vein thrombosis, or pulmonary embolism in the 3 months prior to consent.

Uncontrolled hypertension (≥160/100 mm Hg).

History of or ongoing symptomatic dysrhythmias, uncontrolled atrial or ventricular arrhythmias, or heart block (excluding first-degree block, consisting of PR interval prolongation only). Controlled atrial fibrillation is allowed.

Fridericia heart rate–corrected QT interval (QTcF) > 480 ms.

Severe respiratory illness that significantly impacts functional status in daily life, including known active tuberculosis (*Mycobacterium tuberculosis*).

History of significant weight loss (≥7 kg/15 lbs) within 4 weeks prior to the first dose of study drug.

History of primary immunodeficiency or those with known human immunodeficiency virus, or known active hepatitis B (including core antibody and surface antigen), or hepatitis C infection. Note: No testing for human immunodeficiency virus, hepatitis B, or C is required unless mandated by a local health authority.

Active uncontrolled infection, or an unstable or severe intercurrent medical condition that requires treatment.

History of solid organ transplant or bone marrow transplant.

Any condition or illness that, in the opinion of the investigator, would compromise subject safety or interfere with the evaluation of the safety of the study drug and jeopardise compliance with the protocol and study visits.

Has known active central nervous system metastases and/or carcinomatous meningitis. Note: Subjects with previously treated brain metastases may participate, provided they are radiologically stable, i.e., without evidence of progression for at least 12 weeks by repeat imaging (note that the repeat imaging should be performed during study screening), clinically stable, and without requirement of steroid treatment for at least 14 days prior to the first dose of study treatment.

History of prior malignancy, except for the following: curatively treated basal or squamous cell carcinoma of the skin (nonmelanoma skin cancer); cervical or vaginal intra-epithelial neoplasia; or non-invasive breast cancer in situ at screening. Subjects with other curatively treated malignancies who have had no evidence of metastatic disease and a >2-year disease-free interval may be enrolled after approval by the medical monitor or sponsor designee.

Females who are pregnant, planning to become pregnant, or breastfeeding.

Hypersensitivity to the study drug or excipients (lactose, microcrystalline cellulose, magnesium stearate, and gelatin capsule shell).

## Appendix B

**Table A1:** Summary of plasma pharmacokinetic parameters: QD cohorts.

Dose Cohort	T_max_ (h)		C_max_ (ng/mL)		C_min_ (ng/mL)		AUC_0–last_ (ng·h/mL)		AUC_0–12_ (ng·h/mL)	
	C1D1	C1D21	C1D1	C1D21	C1D1	C1D21	C1D1	C1D21	C1D1	C1D21
**25 mg, QD**										
n	3	3	3	3	–	3	3	3	3	3
Mean	2.0	2.7	32.5	41.9	–	10.3	269.5	461.3	177.3	286.0
SD	0.98	1.12	19.77	15.41	–	6.71	166.21	203.50	108.55	124.15
%CV	49.3	41.7	60.8	36.8	–	65.1	61.7	44.1	61.2	43.4
Median	2.0	2.1	39.6	41.2	–	7.8	302.0	366.0	211.0	232.0
Min	1.0	2.0	10.2	26.8	–	5.2	89.4	323.0	55.9	198.0
Max	3.0	4.0	47.8	57.6	–	17.9	417.0	695.0	265.0	428.0
**AR Factor**	–		1.3		NE		1.7		1.6	
**50 mg, QD**										
n	3	2	3	2	–	2	3	2	3	2
Mean	2.0	1.5	72.9	99.2	–	34.9	594.0	1210.5	382.0	706.0
SD	1.73	0.75	53.95	78.91	–	10.82	396.78	536.69	257.90	362.04
%CV	86.6	49.9	74.0	79.5	–	31.0	66.8	44.3	67.5	51.3
Median	1.0	1.5	65.8	99.2	–	34.9	643.0	1210.5	413.0	706.0
Min	1.0	1.0	22.8	43.4	–	27.2	175.0	831.0	110.0	450.0
Max	4.0	2.0	130.0	155.0	–	42.5	964.0	1590.0	623.0	962.0
**AR Factor**	–		1.4		NE		2.0		1.9	
**100 mg, QD**										
n	3	2	3	2	–	2	3	2	1	0
Mean	0.8	1.9	141.4	436.5	–	151.8	875.3	1796.0	1350.0	–
SD	0.27	1.24	123.60	351.43	–	112.01	975.72	1391.57	NE	–
%CV	33.3	66.0	87.4	80.5	–	73.8	111.5	77.5	NE	–
Median	0.9	1.9	112.0	436.5	–	151.8	460.0	1796.0	1350.0	–
Min	0.5	1.0	35.1	188.0	–	72.6	176.0	812.0	1350.0	–
Max	1.0	2.8	277.0	685.0	–	231.0	1990.0	2780.0	1350.0	–
**AR Factor**	–		3.1		NE		2.1		–	
**125 mg, QD**										
n	3	3	3	3	–	3	3	3	3	3
Mean	2.0	2.0	102.8	147.4	–	33.7	1036.3	1836.0	672.3	1115.7
SD	1.03	1.00	57.95	72.99	–	17.22	566.29	969.78	355.36	574.11
%CV	50.3	50.0	56.3	49.5	–	51.1	54.6	52.8	52.9	51.5
Median	2.1	2.0	123.0	188.0	–	42.0	1280.0	2340.0	877.0	1380.0
Min	1.0	1.0	37.5	63.1	–	13.9	389.0	718.0	262.0	457.0
Max	3.1	3.0	148.0	191.0	–	45.2	1440.0	2450.0	878.0	1510.0
**AR Factor**	–		1.4		NE		1.8		1.7	
**150 mg, QD**										
n	6	5	6	5	–	5	6	5	6	5
Mean	5.5	2.6	155.7	254.2	–	102.8	1608.3	3620.0	945.7	2138.0
SD	9.14	1.93	64.73	127.79	–	38.78	498.09	1614.57	316.20	965.96
%CV	166.8	73.6	41.6	50.3	–	37.7	31.0	44.6	33.4	45.2
Median	2.1	2.0	125.5	269.0	–	89.9	1460.0	3510.0	892.0	2080.0
Min	0.5	1.0	109.0	126.0	–	65.5	1080.0	2060.0	682.0	1190.0
Max	24.0	6.0	277.0	414.0	–	168.0	2500.0	5820.0	1540.0	3390.0
**AR Factor**	–		1.6		NE		2.3		2.3	
**175 mg, QD**										
n	2	0	2	0	–	0	2	0	1	0
Mean	2.0	–	357.0	–	–	–	1576.0	–	1500.0	–
SD	1.32	–	138.59	–	–	–	939.04	–	NE	–
%CV	66.6	–	38.8	–	–	–	59.6	–	NE	–
Median	2.0	–	357.0	–	–	–	1576.0	–	1500.0	–
Min	1.1	–	259.0	–	–	–	912.0	–	1500.0	–
Max	2.9	–	455.0	–	–	–	2240.0	–	1500.0	–

Abbreviations: AR: accumulation ratio; AUC_0–12_: area under the concentration–time curve (AUC) to 12 h; AUC_0–last_: AUC to the last measurable plasma concentration; C: cycle; C_max_: maximum plasma concentration; C_min_: minimum plasma concentration; CV: coefficient of variance; D: day; NE: not estimable; QD: once daily; SD: standard deviation; and T_max_: time to C_max_.

## Appendix C

**Table A2:** Summary of plasma pharmacokinetic parameters: BID cohorts.

Dose Cohort	T_max_ (h)		C_max_ (ng/mL)		C_min_ (ng/mL)		AUC_0–last_ (ng·h/mL)		AUC_0–12_ (ng·h/mL)	
	C1D1	C1D21	C1D1	C1D21	C1D1	C1D21	C1D1	C1D21	C1D1	C1D21
**75 mg, BID**										
n	3	3	3	3	–	3	3	3	3	3
Mean	9.4	3.0	113.8	267.3	–	147.5	1390.0	4423.3	706.0	2290.0
SD	12.78	2.69	75.11	134.34	–	104.08	835.94	2627.17	511.85	1270.00
%CV	136.0	90.6	66.0	50.3	–	70.5	60.1	59.4	72.5	55.5
Median	3.0	1.9	93.4	331.0	–	128.0	1110.0	4300.0	541.0	2290.0
Min	1.1	1.0	51.0	113.0	–	54.6	730.0	1860.0	297.0	1020.0
Max	24.1	6.0	197.0	358.0	–	260.0	2330.0	7110.0	1280.0	3560.0
**AR Factor**	–		2.4		NE		3.2		3.2	
**110 mg, BID**										
n	3	3	3	3	–	3	3	3	3	3
Mean	3.0	2.4	95.2	248.7	–	145.6	1172.3	4280.0	638.7	2260.0
SD	1.04	0.56	29.76	166.31	–	118.78	209.73	3511.58	169.19	1773.05
%CV	34.8	23.8	31.2	66.9	–	81.6	17.9	82.0	26.5	78.5
Median	3.0	2.1	93.1	187.0	–	90.2	1130.0	2560.0	593.0	1390.0
Min	2.0	2.0	66.6	122.0	–	64.7	987.0	1960.0	497.0	1090.0
Max	4.0	3.0	126.0	437.0	–	282.0	1400.0	8320.0	826.0	4300.0
**AR Factor**	–		2.6		NE		3.7		3.5	
**150 mg, BID**										
n	6	6	6	6	–	6	6	6	5	6
Mean	9.0	3.5	235.8	527.0	–	243.5	2328.3	9075.0	1045.2	4615.0
SD	11.66	2.19	143.93	187.52	–	115.05	1372.26	2972.37	523.22	1932.42
%CV	130.1	63.5	61.0	35.6	–	47.2	58.9	32.8	50.1	41.9
Median	2.0	2.9	207.5	549.0	–	242.5	1685.0	9995.0	1070.0	4865.0
Min	0.9	1.0	123.0	249.0	–	114.0	1330.0	5350.0	404.0	2350.0
Max	24.0	6.0	514.0	772.0	–	414.0	4830.0	12,800.0	1810.0	7450.0
**AR Factor**	–		2.2		NE		3.9		4.4	
**200 mg, BID**										
n	3	2	3	2	–	2	3	2	3	2
Mean	9.7	2.4	174.0	874.5	–	471.5	2053.3	16,405.0	985.3	8550.0
SD	12.36	0.64	66.09	318.91	–	314.66	642.99	10,740.95	512.55	4879.04
%CV	127.5	26.3	38.0	36.5	–	66.7	31.3	65.5	52.0	57.1
Median	3.1	2.4	178.0	874.5	–	471.5	2170.0	16,405.0	1130.0	8550.0
Min	2.0	2.0	106.0	649.0	–	249.0	1360.0	8810.0	416.0	5100.0
Max	24.0	2.9	238.0	1100.0	–	694.0	2630.0	24,000.0	1410.0	12,000
**AR Factor**	–		5.0		NE		8.0		8.7	

Abbreviations: AR: accumulation ratio; AUC_0–12_: area under the concentration–time curve (AUC) to 12 h; AUC_0–24_: AUC_0–last_: AUC to the last measurable plasma concentration; C: cycle; C_max_: maximum plasma concentration; C_min_: minimum plasma concentration; CV: coefficient of variance; D: day; NE: not estimable; SD: standard deviation; and T_max_: time to C_max_.
